# Temporal variation in scattering and intrinsic attenuation due to earthquakes in East Asia

**DOI:** 10.1038/s41598-021-90781-8

**Published:** 2021-05-27

**Authors:** Muhammad Zafar Iqbal, Tae Woong Chung, Myung Jin Nam, Kazuo Yoshimoto

**Affiliations:** 1 Department of Energy Resources and Geosystems Engineering , Sejong University, Seoul, 05006 South Korea; 2 Micro Seismic Studies Program, Centre for Earthquake Studies, National Centre for Physics , Islamabad, 46000 Pakistan; 3 Department of Materials System Science, Yokohama City University, Yokohama, 236-0027 Japan

**Keywords:** Natural hazards, Solid Earth sciences

## Abstract

Separated attenuation values have not been used in post-seismic variation research, although the scattering attenuation (*Q*_*s*_^−1^) parameter that can be used to estimate crustal inhomogeneity due to cracks. In this study, three earthquakes that occurred in Kumamoto (*M*7.3), Tottori (*M*6.6), and Gyeongju (*M*5.8) in 2016 were investigated by applying a multiple lapse time window analysis to seismograms recorded before and after the events. At a low frequency, significantly greater variation of the *Q*_*s*_^−1^ value was observed than the intrinsic attenuation (*Q*_*i*_^−1^) for the Kumamoto earthquake, whereas similarly large variation was observed for the Gyeongju earthquake. For the surrounding Kumamoto earthquake area of increased attenuation, even higher decreases in *Q*_*s*_^–1^ and *Q*_*i*_^–1^ were also observed. The increases occurred within a two year-period after mainshock. The large increases in attenuation, corresponding to regions with high peak ground acceleration, were limited to the basin area with an elevation below 500 m. Furthermore, post-seismic increases in attenuation values were found to correlate with the magnitude and length of the quiet periods of the earthquakes. From this study, *Q*_*s*_^–1^ and *Q*_*i*_^–1^ were shown as new parameters that can quantitatively measure the post-seismic deformation due to crustal earthquake.

## Introduction

Temporal variations in the crust related to shallow earthquakes have been widely investigated to understand the behavior of faults and earthquake cycle. For the investigation, velocity changes have been mainly reported for both the occurrence of cracks and their healing^1–3^. Crack-related observation may also be effective by means of anelastic attenuation, expressed as seismic quality factor *Q*, and there are few observations by coda *Q* attenuation^4,5^. However, several studies have also reported that detectible changes were not observable by this method^6–8^.

Total attenuation of *Q*_*t*_, including coda *Q*, is physically separated into scattering and intrinsic attenuations, as shown in the following equation:

1 $$Q_{t}^{ - 1} = Q_{s}^{ - 1} + Q_{i}^{ - 1}$$

where *Q*_*s*_^−1^ is scattering attenuation, and *Q*_*i*_^−1^ is intrinsic attenuation. *Q*_*s*_^−1^ represents the heterogeneity of the medium that redistributes seismic energy without loss and *Q*_*i*_^−1^ represents the anelasticity that converts the wave energy into frictional heat. The parameter *Q*_*i*_^−1^ is closely related to the high temperature caused by igneous activity^9–11^, whereas *Q*_*s*_^−1^ reflects the heterogeneous structure of the medium^12,13^. In particular, observations of depth-dependent *Q*_*s*_^−1^ values reflecting strong inhomogeneity in the upper crust^14,15^ suggest that scattering attenuation can be used to estimate post seismic variations due to earthquake fractures.

**Figure 1:**
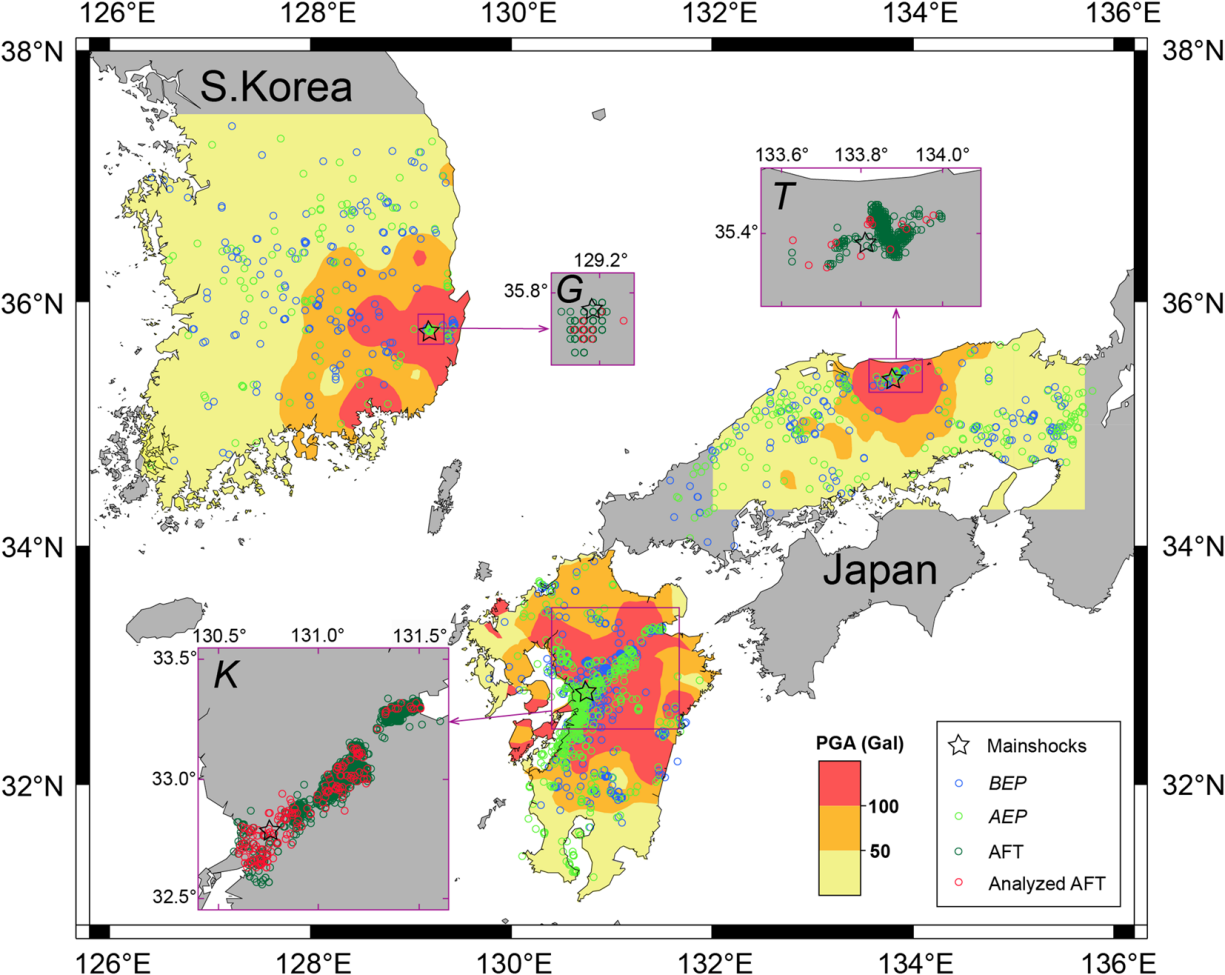
Earthquakes (stars) with aftershocks (green circles in insets) are shown for the Kumamoto (***K***) and Tottori (***T***) earthquakes in Japan and the Gyeongju (***G***) earthquake in Korea in 2016. The analyzed events are separated into the before-earthquake period (*BEP*; blue circles) and after-earthquake period (*AEP*; light-green circles). For the aftershocks (insets), those examined in this study are highlighted by red circles. The study regions are classified based on peak ground acceleration (PGA) values.

The *Q*_*s*_^−1^ and *Q*_*i*_^−1^ values have been effectively obtained by multiple lapse time window analysis^16,17^ (MLTWA) using events with hypocentral distance within approximately 100 km. Based on the MLTWA, the temporal variations in the *Q*_*s*_^−1^ and *Q*_*i*_^−1^ values were compared for three earthquakes that occurred in 2016 (Fig. 1). Specifically, two shallow (~ 10 km) Japanese inland earthquakes with magnitudes (*M*) of 7.3 and 6.6 were selected and a Korean earthquake (*M*5.8) that occurred at a moderate depth (15 km). These earthquakes provided an opportunity to estimate the temporal *Q*_*s*_^−1^ and *Q*_*i*_^−1^ variation related to the earthquake magnitude, if it exists, it could be observed as a velocity variation for the earthquakes^2^. This study newly added parameters that can be used for the quantitative estimation of post-seismic deformations caused by crustal earthquakes.

## Earthquake regions and data

The two Japanese earthquakes, i.e., the Kumamoto (*K*) and Tottori (*T*) earthquakes, occurred at 01:25 Japan standard time (JST) on April 16, 2016, and at 14:07 JST on October 21, 2016, respectively. The South Korean Gyeongju (*G*) earthquake occurred at 20:32 Korea standard time (KST) on September 12, 2016. The moment magnitudes (*Mw*) of events *K*, *T* and *G* are 7.0, 6.2 and 5.4, respectively, which represent smaller values than the local *M*. Figure 1 shows that the large magnitude of the *K* event corresponds to a relatively large area with high peak ground acceleration (PGA) values.

**Figure 2:**
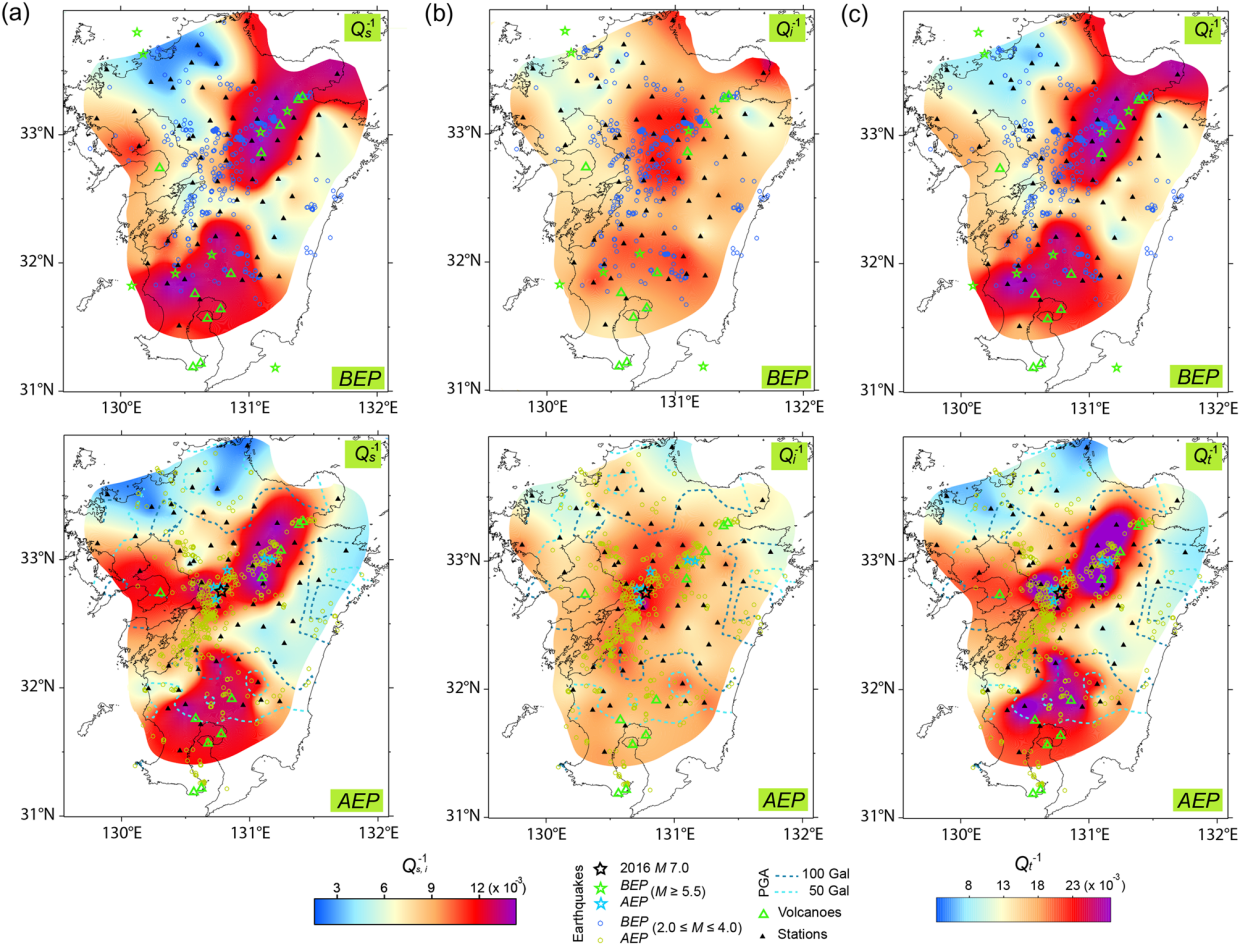
Map of the (**a**) *Q*_*s*_^–1^, (**b**) *Q*_*i*_^–1^, and (**c**) *Q*_*t*_^–1^ value at 1.5 Hz in the *BEP* (top) and *AEP* (bottom) for the Kumamoto earthquake (*M*7.0). Earthquakes (*M* ≥ 5.5) and analyzed events (2.0 ≤ *M* ≤ 4.0) were classified based on the *BEP* and *AEP*. In the *AEP*, blue and sky-blue dotted lines represent a PGA of 100 and 50 Gal (shown in Fig. 1), respectively. Stations (▲) and volcanoes (△) are displayed for both periods.

**Figure 3:**
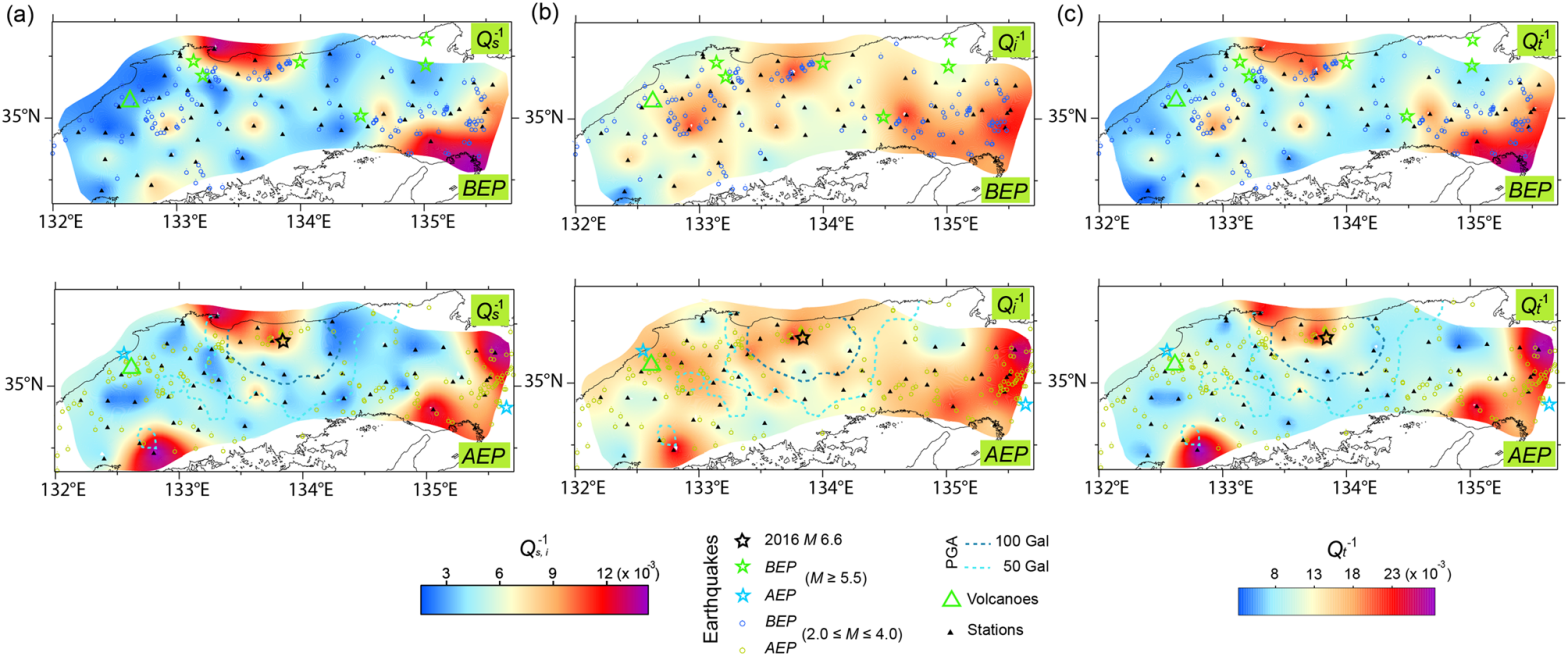
Map of the (**a**) *Q*_*s*_^–1^, (**b**) *Q*_*i*_^–1^, and (**c**) *Q*_*t*_^–1^ value at 1.5 Hz in the *BEP* (top) and *AEP* (bottom) for the Tottori earthquake (*M*6.2). The styles and symbols are the same as those in Fig. 2.

**Figure 4:**
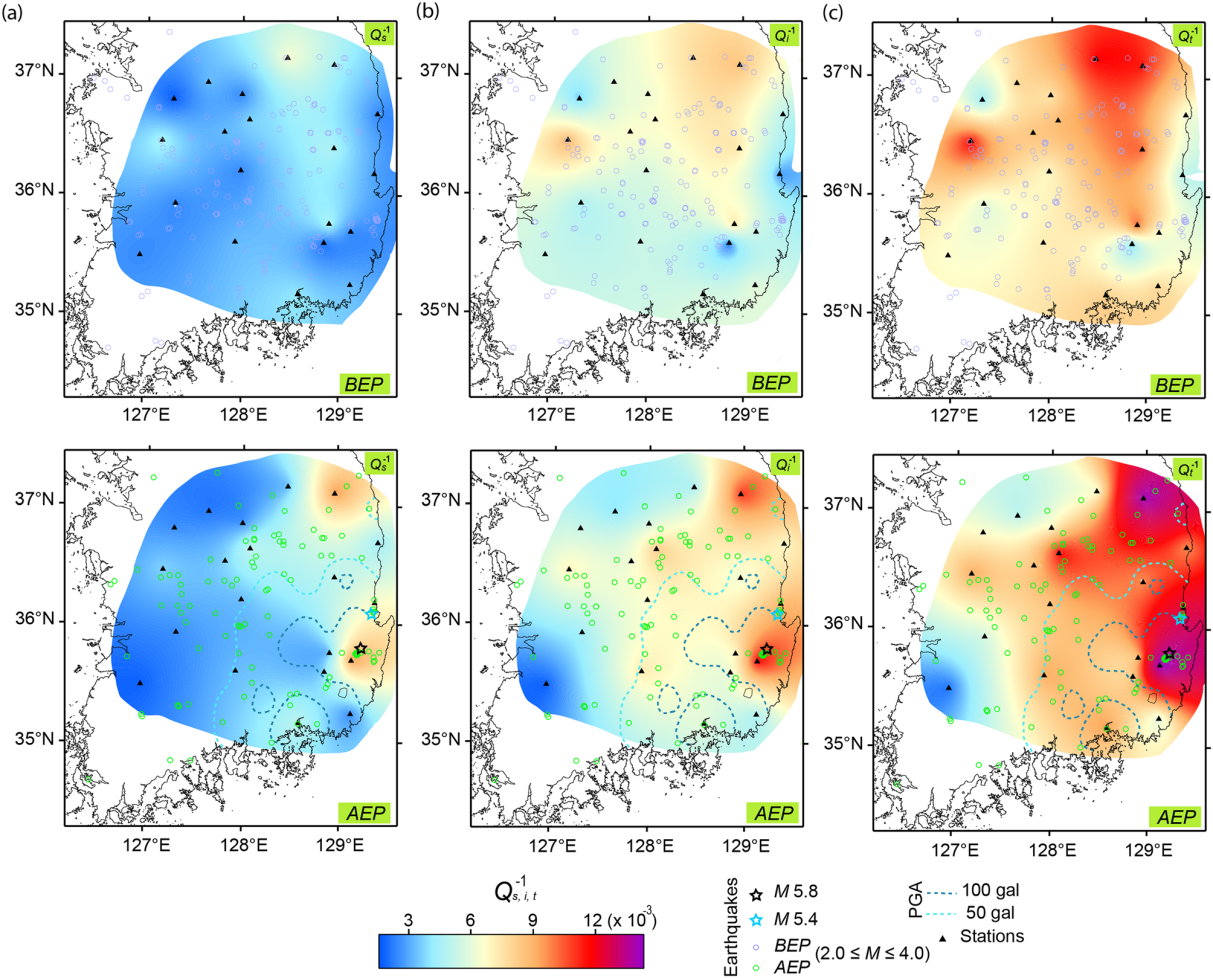
Map of the (**a**) *Q*_*s*_^–1^, (**b**) *Q*_*i*_^–1^, and (**c**) *Q*_*t*_^–1^ value at 1.5 Hz in the *BEP* (top) and *AEP* (bottom) for the Gyeongju earthquake (*M*5.8). The styles and symbols are the same as those in Fig. 2.

In the studied regions, several shallow (< 20 km) inland earthquakes have occurred since 1920 at *M* ≥ 5.5, including *Mw* of up to 5.5 (Figs. 2, 3, 4, see Supplementary Table S1).

Although six earthquakes have been recorded, only one of the earthquakes (*M*6.1 in 1975) occurred close to the location of event *K* (Fig. 2). Six additional earthquakes were also recorded in the region near the location of event *T*, and two large earthquakes (*M*7.0 in 1943 and *M*6.7 in 2000) occurred in the same vicinity (Fig. 3). In contrast, South Korea is relatively seismically stable. From 1905 until event *G*, only three inland events with magnitudes ranging from 5.0 to 5.2 occurred—notably, no inland event at *M* > 5.2 has been recorded for 270 years^18,19^. However, on November 15, 2017, an earthquake (*M*5.4) with a shallow focal depth (< 5 km) did occur (Fig. 4, see Supplementary Table S1), which was more destructive than event *G*^20^.

**Figure 5:**
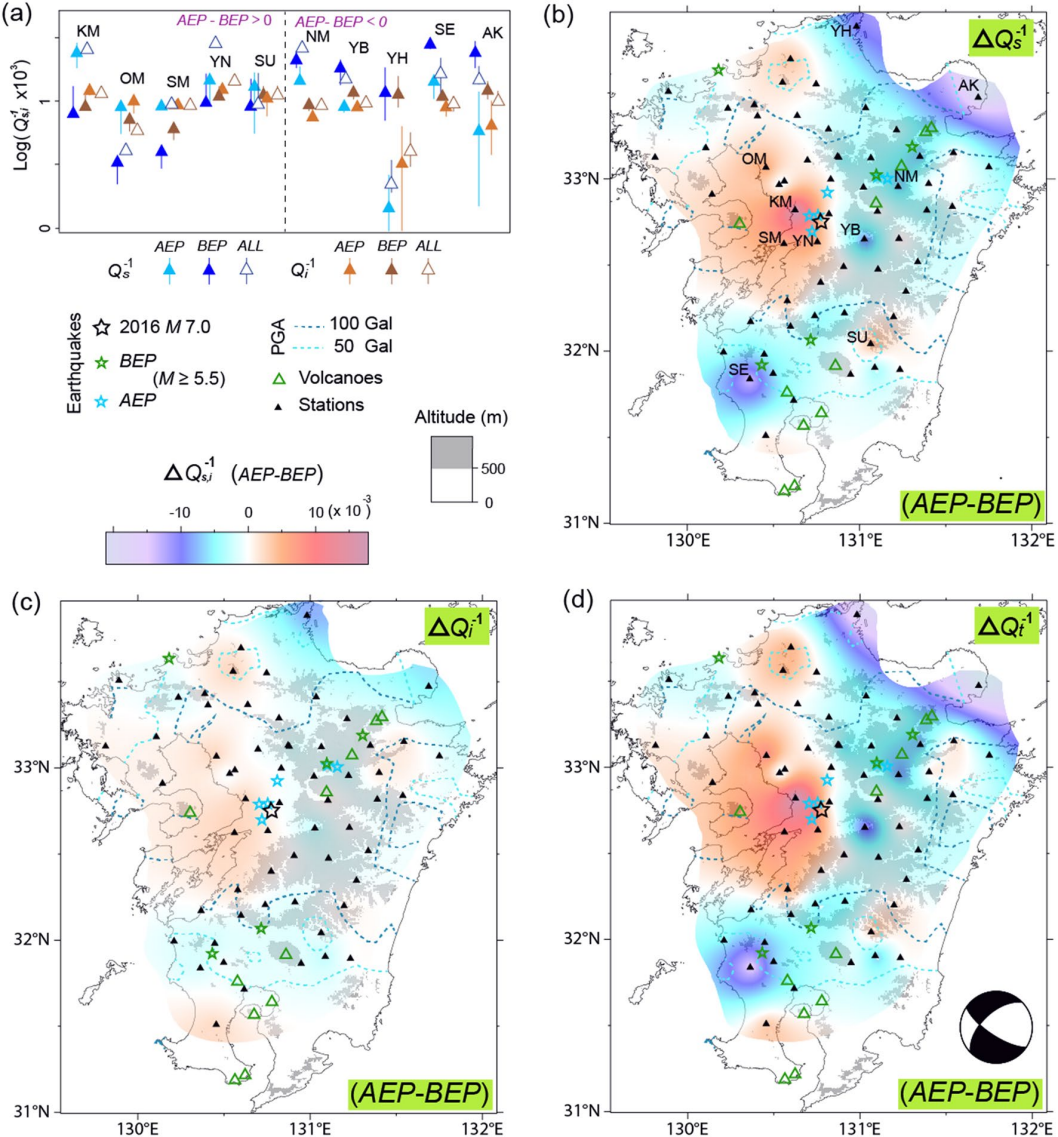
(**a**) The *Q*_*s*_^–1^ and *Q*_*i*_^–1^ value of the *BEP* (blue and brown, respectively) and *AEP* (violet and orange, respectively) are compared for the stations. All (*BEP* + *AEP*) of *Q*_*s*_^–1^ and *Q*_*i*_^–1^ value is also plotted as blue and brown open triangles, respectively. The common logarithm of *Q*_*s*_^–1^ value multiplied by 10^3^, *AEP-BEP*, are divided by dotted line as 5 largest (plus) and 5 smallest (minus) ones. The error bars are based on Fisher’s *F* distribution with a confidence of 60%. (**b**–**d**) Topographic maps showing the difference between the *BEP* and *AEP* (*AEP-BEP*) for the *Q*_*s*_^–1^, *Q*_*i*_^–1^, and *Q*_*t*_^–1^ value, respectively, of the Kumamoto earthquake at 1.5 Hz. Focal mechanism of mainshock in (**d**) is from CMT catalog from Hi-net (https://www.hinet.bosai.go.jp/AQUA/aqua_catalogue.php?LANG=en). The other symbols are the same as those in Fig. 2.

The three earthquakes *K, T*, and *G* were examined using the N–S component velocity seismograms recorded in the before-earthquake period (*BEP*) and after-earthquake period (*AEP*) including the mainshock. The *BEP* and *AEP* were set according to the following event start and end dates to obtain enough data: January 3, 2012, and April 9, 2020, for event *K*; January 4, 2015, and August 31, 2018, for event *T*; and March 13, 2003, and June 8, 2020, for event *G*. Thus, the length of the *BEP* and *AEP* were both 5 years for event *K*, 2 years and 3 years for event *T*, and 14 years and 5 years for event *G*. It is evident that the time range of these events are not affected by the previous major events (Figs. 3, 4, and 5, and Supplementary Table S1). The events in Japan were recorded on Hi-net stations^21^ while those in Korea were recorded by the Korea Meteorological Administration (KMA). The Japanese event information (i.e., origin time, hypocenter, and magnitude) was based on a catalog provided by the Japan Meteorological Agency (JMA), which is available as a refined or preliminary catalog.

To determine the crustal variation associated with the earthquakes, the focal depths and hypocentral distances of the events were limited to those shallower than 30 km and shorter than 80 km, respectively. The magnitudes of the events ranged from 2.0 to 4.0. Based on these criteria, 544 and 633 events were obtained for event *K* (for *BEP* and *AEP*, respectively), 179 and 203 events for event *T*, and 130 and 103 events for event *G*. For the *AEP* events, target events were carefully selected from the aftershock clusters to avoid biased spatial coverage (Fig. 1). All events were recorded at 60, 55 and 20 stations (see Supplementary Fig. S1), respectively. The total number of seismograms obtained in the *BEP* and *AEP* were 10,207 and 10,429 for event *K*, 2624 and 2701 for event *T*, and 595 and 497 for event *G*, respectively. In addition, this study obtained the PGA values of event *G* based on acceleration seismograms of 46 stations with epicentral distance < 150 km (see Supplementary Table S2).

## Results and discussion

The regional differences in *Q*_*s*_^–1^, *Q*_*i*_^–1^, and *Q*_*t*_^–1^ value was most pronounced at 1.5 Hz (Figs. 2, 3, 4) and to a lesser extent at higher frequencies (see Supplementary Figs. S2, S3, S4, and Supplementary Tables S2, S3, S4); while the differences were negligible at 6 Hz. Similar regional differences have been reported by previous studies in Japan^22,23^. Higher *Q*^–1^ value was, however, obtained compared to those by Carcolé and Sato^22^, because their maximum hypocentral distance was 20 km longer than ours. For this study, the shorter distance used for the MLTWA, which reflects shallower values, provided the higher *Q*_*s*_^–1^ and *Q*_*i*_^–1^ values^13^. For the region of event *K*, the high *Q*_*s*_^–1^ and *Q*_*i*_^–1^ value were correlated with an area of active tectonics due to previous large earthquakes and volcanoes (Fig. 2), whereas the low *Q*^–1^ zone was consistent with the low heat flow area^24^. In the surrounding region of event *T*, high *Q*^–1^ value was obtained in the north-central coastal region, corresponding to active tectonics due to previous large events (Fig. 3). A high heat flow near the eastern coast of South Korea^25^ appeared to be related to high *Q*_*i*_^–1^ and *Q*_*t*_^–1^ values of the region around event *G* (Fig. 4).

For the regions of events *K* and *T*, *Q*_*s*_^–1^ had a relatively similar distribution to *Q*_*t*_^–1^ compared to *Q*_*i*_^–1^. The high *Q*_*s*_^–1^ distribution related to event *K* was more pronounced than *Q*_*t*_^–1^ along the western coast for the *AEP* (Fig. 2).

For the *AEP* of event *G*, both the *Q*_*s*_^–1^ and *Q*_*i*_^–1^ value contributed to the high *Q*_*t*_^–1^ value in the seismic area (Fig. 4). In addition to a high heat flow area, the high PGA areas of events *K* and *G* corresponded to regions with high *Q*_*s*_^–1^, *Q*_*i*_^–1^, and *Q*_*t*_^–1^ value. Figure 5a shows the five stations with largest variations at 1.5 Hz for both *AEP-BEP* and *BEP-AEP*, while, including all (*AEP* + *BEP*) data. Values of *Q*_*s*_^–1^ and *Q*_*i*_^–1^ for all stations except YN and of *Q*_*s*_^–1^ for all stations except NM are located between *AEP* and *BEP* with shorter error bars. The five largest increase of *Q*_*s*_^–1^, ranging from 0.004 to 0.016, were mainly distributed in the basin area with elevations below 500 m, and close to the epicenter of *K* (Fig. 5b). However, for the basin area in the eastern coast of event *K* region showing high PGA, stations could not be analyzed because data of very few events were available (≤ 10).

**Figure 6:**
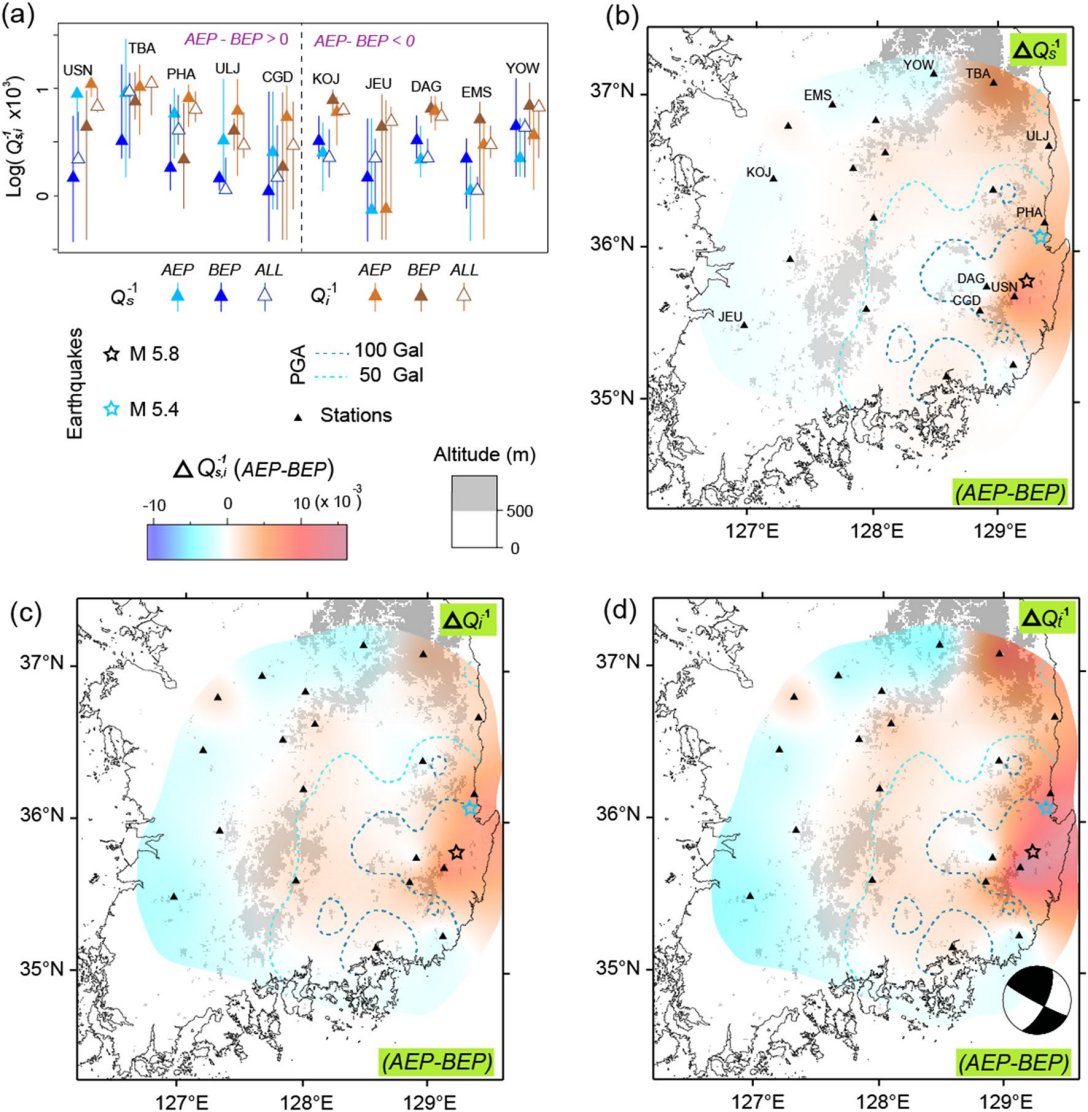
(**a**) The *Q*_*s*_^–1^ and *Q*_*i*_^–1^ value of the *BEP, AEP*, and all are compared for the stations. (**b**–**d**) Topographic maps showing the difference between the *BEP* and *AEP* (*AEP*–*BEP*) for the *Q*_*s*_^–1^, *Q*_*i*_^–1^, and *Q*_*t*_^–1^ value, respectively, of the Gyeongju earthquake at 1.5 Hz. Focal mechanism^28^ of mainshock are shown in (**d**). The styles and symbols are the same as those in Fig. 5.

The corresponding increase of *Q*_*i*_^–1^ was observed in the basin area with values up to 0.003. For the extensive region surrounding the basin area, even higher decreases of *Q*_*s*_^–1^ and *Q*_*i*_^–1^ compared to the increases in *Q*_*s*_^–1^ and *Q*_*i*_^–1^ were observed, ranging from 0.007 to 0.019 and 0.002 and 0.008, respectively. However, several high values (i.e., those at stations AK and YH) showed large error bars due to the small number of observation data. For event *T*, notable variations of *Q*_*s*_^–1^ and *Q*_*i*_^–1^ value did not appear to be related to the location of the epicenter (see Supplementary Fig. S5). However, event *G* showed variation associated with the epicenter, with similarly large values of *Q*_*s*_^–1^ and *Q*_*i*_^–1^, ranging from − 0.002 to 0.008, and from − 0.003 to 0.007, respectively (Fig. 6a). Values of *Q*_*s*_^–1^ and *Q*_*i*_^–1^ for *AEP* + *BEP* were also found to be between those for *AEP* and *BEP*, except at ULJ and JEU (represented by shorter error bars). The increased *Q*_*i*_^–1^ value may have been due to fluid-filled cracks formed during post-seismicity^26,27^.

**Figure 7:**
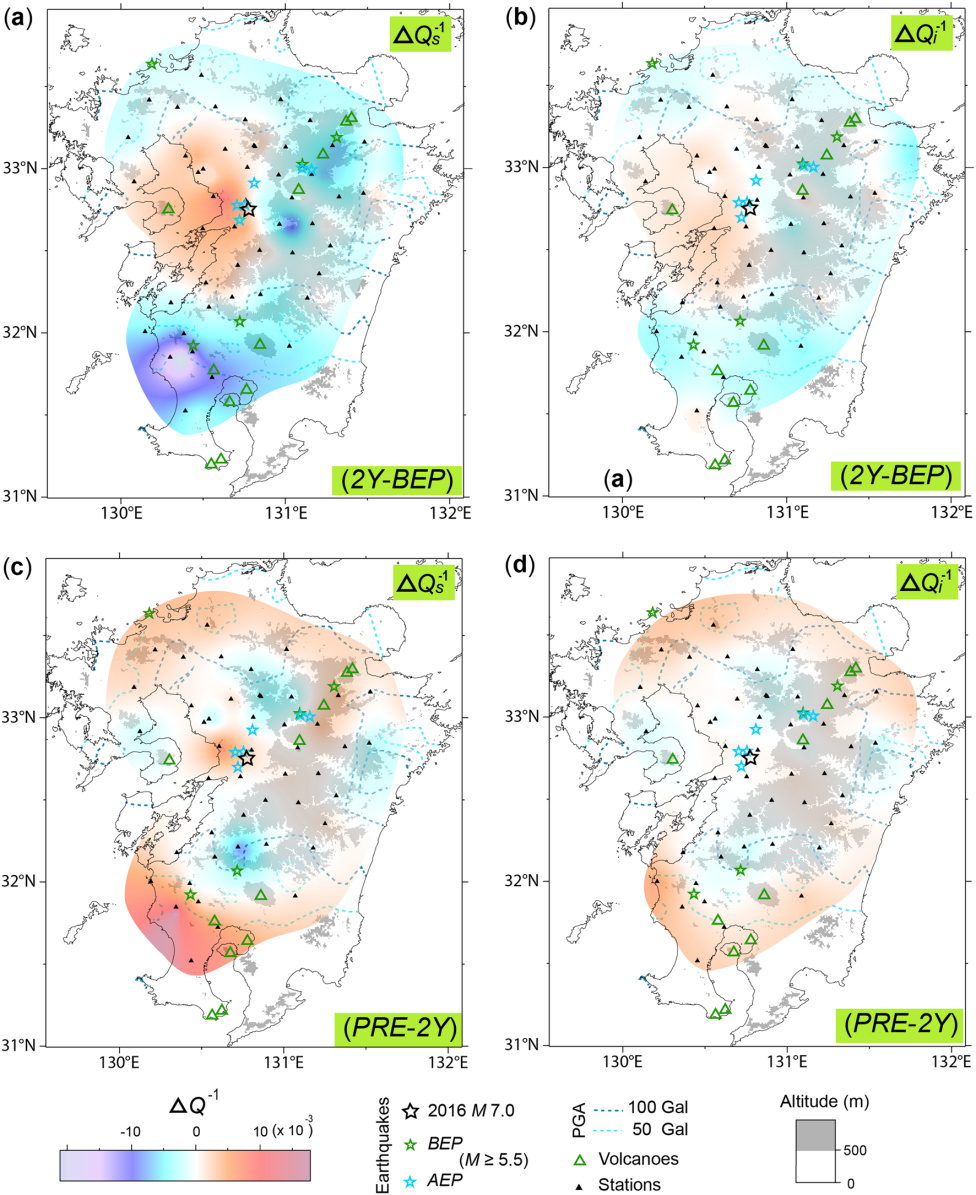
Topographic maps of the Kumamoto earthquake illustrating the difference between the two-year period after the event (*2Y*) and the *BEP* (*2Y-BEP*) for (**a**) the *Q*_*s*_^–1^ and (**b**) *Q*_*i*_^–1^ value, respectively. Topographic maps for the values of (**c**) *Q*_*s*_^–1^ and (**d**) *Q*_*i*_^–1^ reveal the difference between the present (*PRE*) and the *2Y* (*PRE-2Y*), respectively. The styles and symbols are the same as those in Fig. 5.

For the basin area of events *K* and *G*, a large increase in the *Q*_*s*_^–1^ and *Q*_*i*_^–1^ value corresponded to areas with a PGA ≥ 100 Gal, including some stations with a PGA ≥ 50 Gal. The only exception was high values in the northern area caused by station TBA in event *G*, which may have been a result of the large errors in *BEP* (Fig. 6a). The increase in *Q*_*s*_^–1^ and *Q*_*i*_^–1^ in the basin is easily explained as increased cracks, which has also been reported by several post-seismic monitoring studies^29,30^. The sufficient level of data enabled event *K* to be divided into two periods, separated by two years, and this indicated that the *Q*_*s*_^–1^ and *Q*_*i*_^–1^ increases occurred in the first period (Fig. 7a,b see Supplementary Table S6).

In this study, the extensive post-seismic decrease in *Q*_*s*_^–1^ and *Q*_*i*_^–1^ was found surrounding the region of increased *Q*_*s*_^–1^ and *Q*_*i*_^–1^. A similar distribution of decrease was also observed for the first period. The decrease in *Q*_*t*_^–1^ along the earthquake fault was explained as compressional stress related to direction of strike-slip fault movement^30^. However, directional correlation with focal mechanism was not observed in the zone with decreased *Q*_*t*_^–1^ in event *K* and *G* (Figs. 5, 6). The previous research of post-seismic *Q*_*t*_^–1^ was, however, limited to the narrow fault region. The decrease in *Q*_*s*_^–1^ and *Q*_*i*_^–1^ value indicates the rebound phase of increase because the region of increase and decrease of *Q*_*s*_^–1^ and *Q*_*i*_^–1^ value appeared to be reversed between the first and second periods (Fig. 7, see Supplementary Table S6). For the explanation of the rebound phase, further research is required for regional post-seismic *Q*_*s*_^–1^ and *Q*_*i*_^–1^ variation for major earthquakes, as other geophysical parameters have shown extensive variation^31–34^.

Despite the smallest magnitude of the three events, a relatively large difference was obtained adjacent to the area of event *G* (Fig. 6). The two earthquakes in Japan may have been affected by previous earthquakes that occurred near that event (Figs. 2, 3). In contrast, more than 300 years had passed without an earthquake with the same estimated magnitude as event *G* in its vicinity^19^. Thus, the duration of the seismically-quiet period is strongly correlated with the difference in the *Q*_*s*_^–1^ value between the *BEP* and *AEP*. Owing to this low seismicity, an event with *M*5.4 may have caused large post-seismic variation outside the area with high PGA, mainly in the region of the northern coastal basin.

Just like monitoring seismic velocities, this study confirms that a monitoring approach of *Q*_*s*_^–1^ and *Q*_*i*_^–1^ is also crucial for quantitative estimation of the post-seismic variations for crustal earthquakes.

## Methods

**Figure 8:**
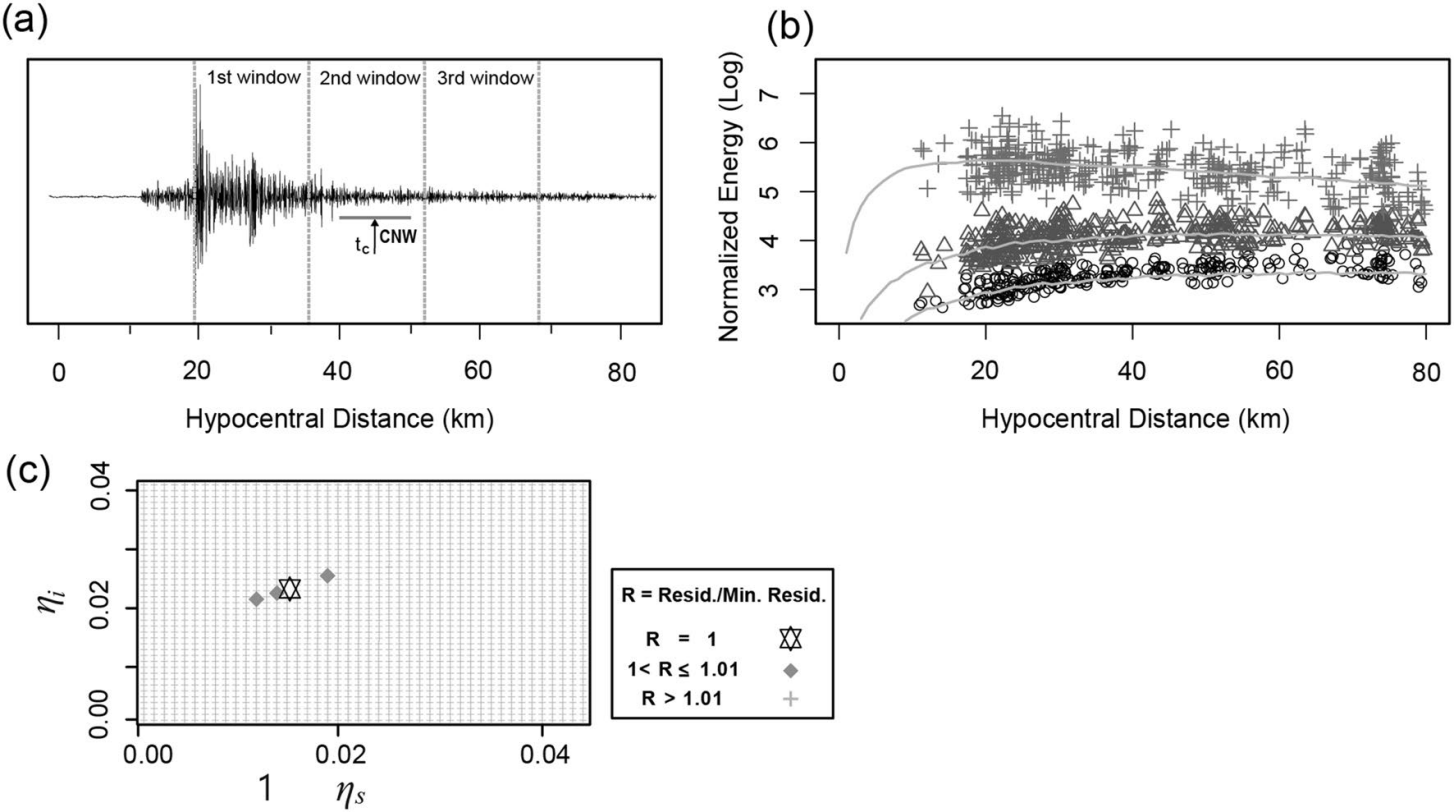
**(a)** Example of seismogram (N–S component) used for multiple lapse time window analysis (MLTWA) showing three-time windows (0–15 s, 15–30 s, and 30–45 s) starting at the arrival of *S*-waves. The time bar with 10 s denotes the coda normalization window (CNW). Its center (*t*_*c*_) denotes a fixed reference time (45 s lapse time). (**b**) Example of a MLTWA fit of the normalized energy recorded of the Kumamoto earthquake in the 1–2 Hz frequency band. The theoretical curves based on the plus signs, triangles, and circles obtained from the first, second, and third windows in the left diagram, respectively, were fitted using Monte Carlo simulations^33^. (**c**) Residual map corresponding to (b). The areas normalized by the minimum residual value (star), = ${{\varvec{M}}}_{{\varvec{f}}}\left({{\varvec{\eta}}}_{{\varvec{s}}},{{\varvec{\eta}}}_{{\varvec{i}}}\right)/{{\varvec{M}}}_{{\varvec{f}}}\left(\widehat{{{\varvec{\eta}}}_{{\varvec{s}}}},\widehat{{{\varvec{\eta}}}_{{\varvec{i}}}}\right)$1.01, are plotted with dark rhombuses as the *F* test confidence zone at the 60% level.

MLTWA, as a method of data analysis, separated all the seismograms into three successive time windows (15 s) after the arrival of the *S*-wave (Fig. 8a). The energy contained in each window of an observation *k* were integrated and called *O*_*k,j*_ where *j* = 1, 2, 3 indicates the window. Data with signal-to-noise ratios above 2 were selected by estimating the noise 5 s before the arrival of the *P*-wave.

After generating bandpass-filtered seismograms with four frequencies centered at 1.5, 3, 6, and 12 Hz, the seismic energy of the three-time windows was obtained from the integrated values of the squared amplitudes over time. Each integral was normalized with the amplitudes of the coda spectra in the 10 s time window centered at 45 s for the correction of the different earthquake sources and varying site amplification^35^ with normalization factor of *O*_*k,*4_. In addition, the geometrical spread of the energy was corrected by multiplication with a factor of 4*πr*_*k*_^2^, where *r*_*k*_ represents the hypocentral distance.

The observed values were fitted with energy curves (Fig. 8b) obtained from theoretical values derived from multiple scattering models. The theoretical values were based on the numerical approach known as the direct-simulation Monte Carlo (DSMC) method^36^ with a focal depth of 10 km^13^. The DSMC method calculated energy density in three-dimensional media with uniform coefficient of scatterings of *η*_*s*_ = $\frac{2\pi f}{v}$ *Q*_*s*_^*–*1^ and *η*_*i*_ = $\frac{2\pi f}{v}$ *Q*_*i*_^*–*1^, where *v* is the *S*-wave velocity (i.e., 3.5 km/s) and *f* is the frequency. This calculation is based on the mean free path in molecular collisions^37^ of particles randomly moving for the spherical coordinate space. The non-isotropic scattering in early coda^38,39^ was neglected here based on a study that demonstrated its slight effect on MLTWA^40^.

The best fit of *η*_*s*_ and *η*_*i*_ were obtained with a grid search using every 0.001 km^−1^ for the following equation:

2 $$M_{f} \left( {\eta_{s} ,\eta_{i} } \right) = \sum\limits_{k = 1}^{N} {\sum\limits_{j = 1}^{3} {\left[ {\log \left( {4\pi r_{k}^{2} \frac{{O_{k,j} }}{{O_{k,4} }}} \right) - \log \left( {4\pi r_{k}^{2} \frac{{D_{k,j} \left( {\eta_{s} ,\eta_{i} } \right)}}{{D_{k.4} \left( {\eta_{s} ,\eta_{i} } \right)}}} \right)} \right]^{2} } } ,$$

where ${D}_{k,j}\left({\eta }_{s},{\eta }_{i}\right)$ and ${D}_{k.4}\left({\eta }_{s},{\eta }_{i}\right)$ are theoretical integrals for each window corresponding to the observations, ${O}_{k,j}$ and ${O}_{k,4}$. The standard errors of *η*_*s*_ and *η*_*i*_ were evaluated by plotting the confidence contour using *F* distribution test^41^:

3 $$M_{f} \left( {\eta_{s} ,\eta_{i} } \right) = M_{f} \left( {\widehat{{\eta_{s} }},\widehat{{\eta_{i} }}} \right)\left[ {1 + \frac{p}{n - p}F_{60} \left( {p,n - p} \right)} \right],$$

where ${M}_{f}\left(\widehat{{\eta }_{s}},\widehat{{\eta }_{i}}\right)$ is the minimum value of ${M}_{f}\left({\eta }_{s},{\eta }_{i}\right)$, $p \left(=2\right)$ is the number of parameters (${\eta }_{s} {\mathrm{and} \eta }_{i})$, *n* is the observation number, and *F*_60_ is the Fisher distribution function with a confidence level at 60%. The ratios ${M}_{f}\left({\eta }_{s},{\eta }_{i}\right)/{M}_{f}\left(\widehat{{\eta }_{s}},\widehat{{\eta }_{i}}\right)$ were plotted as the confidence area by the shaded zones (Fig. 8c).

For the *BEP, AEP*, *26M*, and *PRE*, the reliable *Q*_*s*_^–1^, *Q*_*i*_^–1^, and *Q*_*t*_^–1^ value for each station were obtained with number of observations over 28, 15, and 9 for the region of event *K*, *T*, and *G*, respectively (see Supplementary Tables S3, S4, S5). Owing to the relatively sparse distribution of stations, the spatial variation of attenuation was roughly estimated by interpolation using Surfer 18 (Golden Software, Inc., USA), instead of high resolution approach^42,43^.

## Supplementary Information

Supplementary Information.

Supplementary Tables.

## Data Availability

The information on Japanese events were obtained from the Japan Meteorological Agency (JMA) earthquake catalog, in a refined form (http://www.data.jma.go.jp/svd/eqev/data/bulletin/hypo_e.html, last accessed September 2020). Including a preliminary form of events information, waveforms of Hi-net, operated by the National Research Institute for Earth Science and Disaster Resilience, Japan, were downloaded from the website (http://www.hinet.bosai.go.jp, last accessed September 2020). Korean seismic waveform data were requested with a preauthorized account from National Earthquake Comprehensive Information System operated by Korea Meteorological Administration (KMA). The earthquake catalogue and Korean waveform data used in this study are listed in Chung and Iqbal (2020) (10.5281/zenodo.4059037). Peak ground amplitude for the Kumamoto and Tottori earthquakes was derived from the Headquarters for Earthquake Research Promotion (https://www.static.jishin.go.jp/resource/monthly/2016/2016_kumamoto_3.pdf) and the Earthquake Research Institute at the University of Tokyo (http://www.eri.u-tokyo.ac.jp/en/2016/10/25/21st-october-2016-earthquake-in-tottori-prefecture/), respectively.
