# Supplementary material for: Temporal variation in scattering and intrinsic attenuation due to earthquakes in East Asia
Source: Sci Rep. 2021 May 27;11:11260. doi: 10.1038/s41598-021-90781-8 (PMC8159974; doi:10.1038/s41598-021-90781-8)
Supplement: Supplementary file 1 — Supplementary Information. [file 41598_2021_90781_MOESM1_ESM.pdf]

Supplementary information for

Temporal Variations in Scattering and Intrinsic Attenuation due to Earthquakes in East Asia

Muhammad Zafar Iqbal[1], Tae Woong Chung[1,*], Myung Jin Nam[1], and Kazuo Yoshimoto[2]

*Corresponding author: chungtw@sejong.ac.kr

[1] Department of Energy & Mineral Resources Engineering, Sejong University, Seoul 05006, Korea

[2] Department of Materials System Science, Yokohama City University, Yokohama 236-0027, Japan

Content of this file

Table S1, S2 and S6; Figs. S1, S2, S3, S4, and S5

Table S3, S4 and S5 are separated files.

Table S1. Large events (M ≥ 5.5) that occurred in the region of the Kumamoto (K), Tottori (T), and Gyeongju (G) earthquake.

Date (y-m-d)	Time (h:m:s)	Lat	Lon	Depth (km)	*M*	Region
1925-05-23	11:09:45	35.423	135.007	15.0	6.6	*T*
1927-03-07	18:27:42	35.632	135.013	10.0	7.0	*T*
1943-09-10	17:37:00	35.448	133.993	15.0	7.0	*T*
1961-05-07	21:14:17	35.016	134.482	15.0	5.6	*T*
1968-02-21	10:44:54	32.066	130.712	10.0	6.2	*K*
1975-01-23	23:19:15	33.026	131.092	10.0	5.8	*K*
1975-04-21	02:35:50	33.190	131.300	7.0	6.1	*K*
1991-08-28	10:29:02	35.338	133.208	12.7	5.5	*T*
1997-03-26	17:31:47	31.920	130.429	10.0	6.1	*K*
1997-04-03	04:33:22	31.824	130.089	10.0	5.5	*K*
2000-06-25	15:34:43	31.183	131.206	10.0	6.0	*K*
2000-10-06	13:30:19	35.456	133.134	10.0	6.7	*T*
2005-03-20	10:53:42	33.807	130.131	10.0	6.6	*K*
2005-04-20	06:11:28	33.637	130.179	18.7	5.5	*K*
2016-04-14	21:26:36	32.788	130.704	9.0	6.2	*K*
2016-04-15	00:03:47	32.697	130.720	8.0	6.0	*K*
2016-04-16	01:25:06	32.791	130.754	10.0	7.3	*K*
2016-04-16	01:45:58	32.924	130.809	10.0	5.7	*K*
2016-04-16	03:55:05	33.005	131.157	13.2	5.5	*K*
2016-04-18	20:42:00	33.014	131.099	10.5	5.5	*K*
2016-09-12	20:32:54	35.760	129.190	14.8	5.8	*G*
2016-10-21	14:07:23	35.374	133.809	5.6	6.2	*T*
2017-11-15	02:29:31	36.120	129.360	4.5	5.4*	*G*
2018-04-09	01:32:31	35.259	132.553	10.3	5.7	*T*
2018-06-18	07:58:35	34.825	135.639	10.3	5.5	*T*

5.4* denotes Mw=5.5

Table S2. Peak Ground Ampitude (PGA) for stations (see Supplementary Fig. S1**c**) around Gyeongju earthquake with epicentral distances.

Stations	Lat	Lon	Epicent Dist	PGA (cm/s^2)			
				EW	NS	UD	3-comp
USN	35.70	129.12	9.2	214	429	410	519.9
DAG	35.77	128.90	26.2	49	61	53	72.5
YC	35.98	128.95	32.7	37	56	46	65.4
YS	35.47	129.08	33.7	13	13	5	13.3
PHA	36.19	129.37	50.5	25	28	15	34.5
MI	35.49	128.74	50.6	42	34	36	45.6
DA	35.89	128.62	53.4	184	125	46	197.0
BUS	35.25	129.11	57.2	37	58	54	68.4
CH	35.54	128.49	67.8	52	13	57	63.4
CS	36.39	129.08	70.7	61	38	107	107.7
BS	35.10	129.03	74.8	52	25	31	53.8
ADO	36.41	128.95	75.4	43	43	33	55.6
CI	36.04	128.38	79.3	9	9	17	17.5
ES	36.36	128.68	80.9	8	8	7	10.0
MAS	35.17	128.57	86.4	33	147	217	262.5
YD	36.53	129.41	87.9	11	6	16	17.5
HN	35.57	128.17	94.5	17	20	32	35.9
ER	35.32	128.28	95.8	7	6	5	8.7
GU	36.23	128.29	96.4	23	72	67	74.8
YE	36.63	129.09	97.2	5	11	10	13.7
AD	36.57	128.70	100.2	16	16	14	18.9
GI	36.08	128.10	104.4	53	40	71	82.5
HC	35.41	128.10	106.0	29	30	16	36.4
ULJ	36.70	129.41	106.4	6	7	5	7.9
KCH	35.61	127.92	115.9	11	36	31	39.1
SJ	36.41	128.16	117.4	13	24	24	29.9
CPR	36.22	127.97	121.1	6	8	5	8.2
TO	34.85	128.44	121.9	18	14	21	25.5
JI	35.16	128.04	123.7	34	27	20	36.1
SA	35.41	127.88	124.7	45	64	36	66.9
DS	35.89	127.77	128.8	7	5	6	8.7
HM	35.51	127.75	133.1	4	4	5	5.0
CY	36.94	128.91	133.6	2	3	2	3.2
YJ	36.87	128.52	137.2	3	3	4	5.1
UJ	36.99	129.41	138.2	48	31	39	48.2
MGY	36.65	128.06	141.7	4	3	2	4.5
OK	36.35	127.79	141.9	5	6	5	7.1
HD	35.08	127.77	149.2	3	39	43	48.8

Table S6. Difference in Q^{-1} values between the *AEP* and *BEP (AEP-BEP),* between the two-year period after the event (*2Y*) and the *BEP* (*2Y-BEP*), and between the present (*PRE*) and the *2Y* (*PRE-2Y*) for the Kumamoto earthquake. The values are arranged in order of the *AEP-BEP* of Q_s^{-1} values.

Station	Q_s^{-1} (×10^{-3})			Q_i^{-1} (×10^{-3})		
	AEP-BEP	*2Y-BEP*	*PRE-2Y*	*AEP-BEP*	*2Y-BEP*	*PRE-2Y*
KM	16.34	10.40	7.80	2.97	2.23	0.74
OM	5.94	5.20	0.74	2.97	2.60	0.37
SM	5.20	5.20	0.00	3.34	3.34	0.00
YN	4.83	4.83	0.00	1.49	1.49	0.00
AS	3.34	3.34	0.00	2.23	2.23	0.00
TK	2.60	2.60	-2.60	0.74	1.11	-1.86
UI	1.86	-1.11	3.71	2.97	-0.37	3.71
ZU	1.11	-3.34	9.66	2.60	1.11	3.34
KH	0.74	0.74	2.23	-0.74	-0.74	0.74
NR	0.74	0.74	-0.74	0.74	0.74	-1.11
GK	0.74	0.74	0.37	0.00	0.00	0.37
HS	0.37	-0.74	1.11	-0.37	-1.86	0.74
NT	0.37	0.37	0.00	1.49	0.74	1.86
IZ	0.37	0.74	-0.37	0.00	0.00	0.00
CH	0.00	0.00	-2.23	-0.37	-0.37	-1.11
HK	0.00	0.00	0.00	0.74	1.11	-0.74
IK	0.00	0.00	-0.37	0.74	0.74	-0.37
MS	0.00	-1.86	1.86	-0.37	-0.74	0.37
NH	0.00	3.34	-3.34	0.74	2.23	-1.11
SR	0.00	-2.23	2.23	0.74	0.00	0.74
UM	-0.74	1.86	-3.34	0.00	1.49	-2.23
RH	-0.74	-0.74	-0.74	0.37	0.37	-0.37
FJ	-0.74	-0.74	2.97	-1.86	-1.49	3.34
SB	-1.11	-1.11	0.74	-1.11	-1.11	0.74
MN	-1.49	-1.11	-0.37	-1.11	-1.11	0.37
NA	-1.49	-1.49	1.49	-0.74	-0.74	1.49
OI	-1.86	-1.86	-1.49	-0.74	-0.74	-1.11
TI	-1.86	2.23	-4.08	-0.74	0.74	-1.49
UK	-2.23	0.37	-2.60	-0.37	0.74	-1.49
MR	-2.23	-2.23	-1.11	-0.74	-1.11	-0.37
AV	-2.23	-2.23	0.00	-0.74	-0.74	0.37
EH	-2.23	-2.23	—	-0.74	-0.74	—
MM	-2.60	-2.60	0.00	-0.37	0.00	-0.37
AI	-3.34	-9.66	6.31	-1.11	-2.97	1.49
YG	-3.34	-3.34	1.86	-1.49	-1.11	0.37
US	-3.71	-7.06	2.23	-1.11	-5.94	3.34
AN	-3.71	-5.57	5.57	-1.49	-4.46	5.94
OG	-3.71	-3.34	-2.97	-0.74	-0.74	-0.74
UW	-3.71	-4.83	-0.74	-1.49	-1.86	-0.37
TD	-4.08	-4.08	0.00	-1.11	-1.49	0.37
ZA	-4.46	-5.20	1.49	-2.97	-4.83	2.97
NK	-4.46	0.00	-4.46	-1.86	0.37	-2.23
HY	-4.83	0.00	-9.28	-0.74	0.37	-2.23
YJ	-5.20	-5.20	0.00	-2.23	-1.86	-0.37
DH	-5.94	-8.17	2.23	-0.37	-0.74	0.37
NM	-6.68	-6.68	3.34	-1.86	-1.86	0.74
SN	-6.68	-6.68	3.34	-0.37	-0.37	0.74
YB	-9.28	-10.40	1.11	-2.60	-3.34	1.11
SE	-14.11	-20.42	10.77	-1.86	-3.71	2.60

—: number of observations < 30

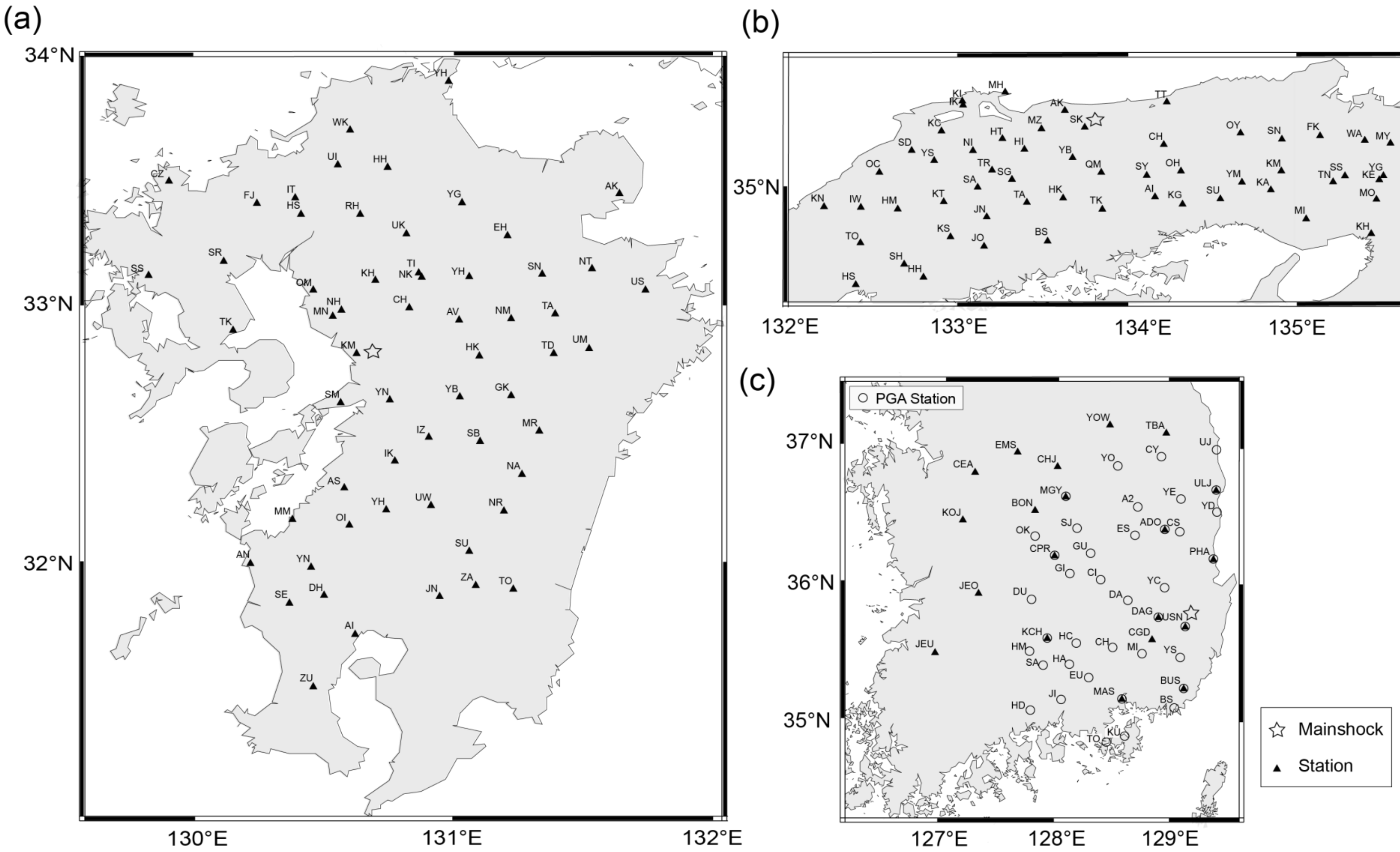

Figure S1. Map of stations for the study area; (a) Kumamoto earthquake (b) Tottori earthquake (c) Gyeongju earthquake. Open circles denote stations for PGA (Table S2)

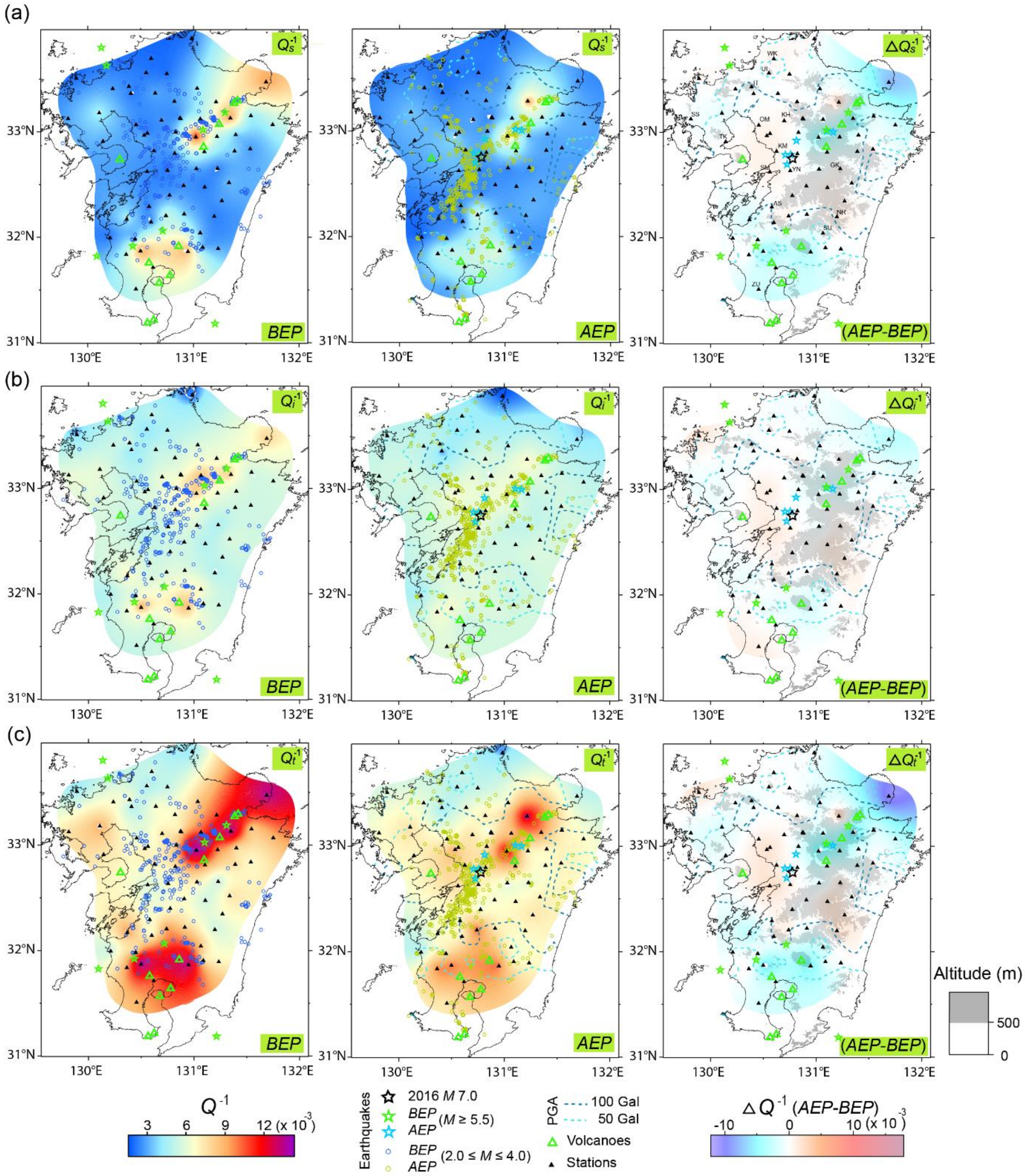

Figure S2. Maps of the (a) Q_s^{-1}, (b) Q_i^{-1}, and (c) Q_t^{-1} values at 3 Hz for *BEP* (left), *AEP* (center), and the difference between the *BEP* and *AEP* (*AEP-BEP*) (right) for the Kumamoto earthquake. The symbols and styles are the same as those in Figures 2.

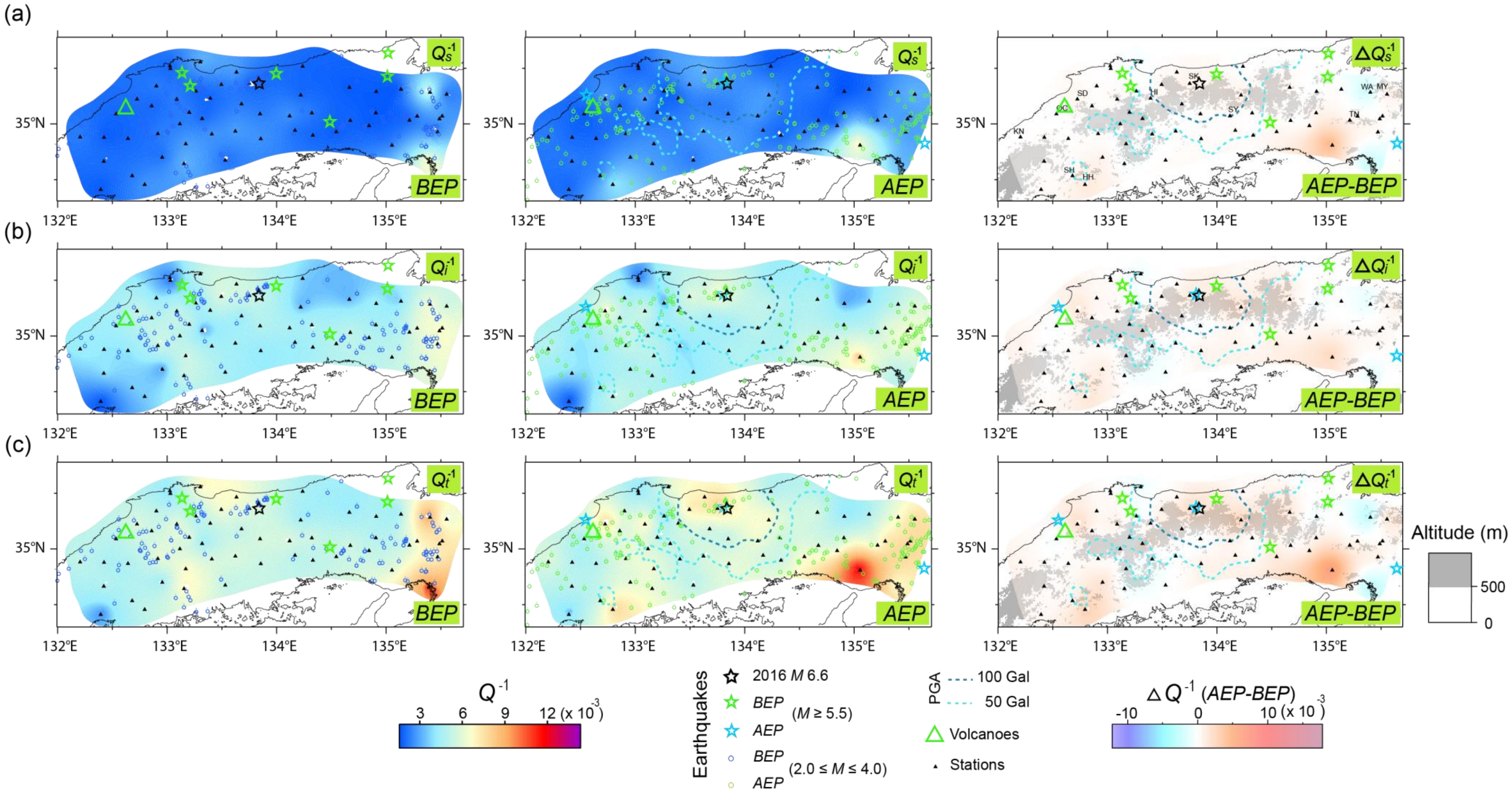

Figure S3. Maps of the (a) Q_s^{-1}, (b) Q_i^{-1}, and (c) Q_t^{-1} values at 3 Hz for *BEP* (left), *AEP* (center), and *AEP-BEP* (right) for the Tottori earthquake. The symbols and styles are the same as those in Figure 2.

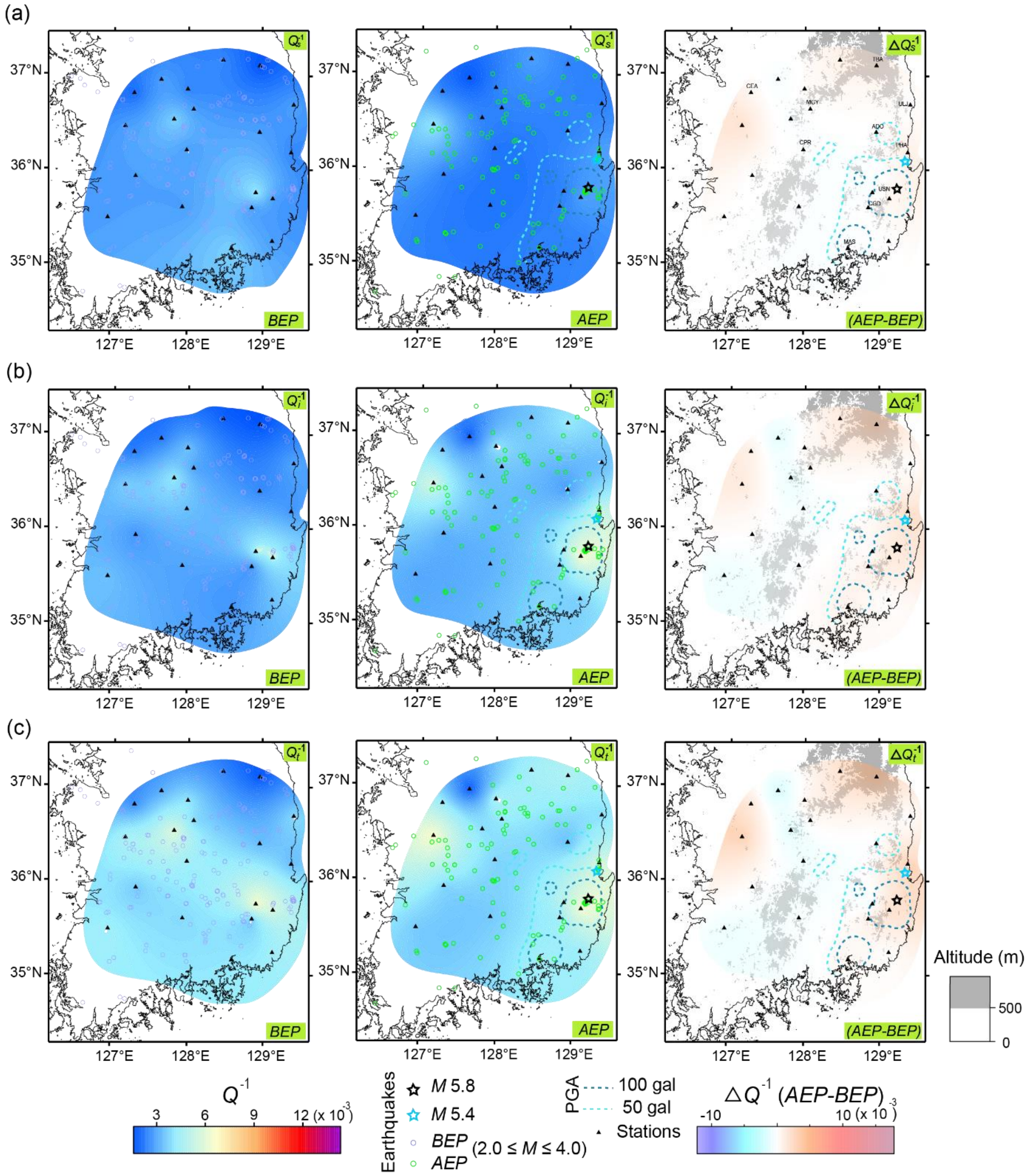

Figure S4. Maps of the (a) Q_s^{-1}, (b) Q_i^{-1}, and (c) Q_t^{-1} values at 3 Hz for *BEP* (left), *AEP* (center), and *AEP-BEP* (right) for the Gyeongju earthquake. The symbols and styles are the same as those in Figures 2.

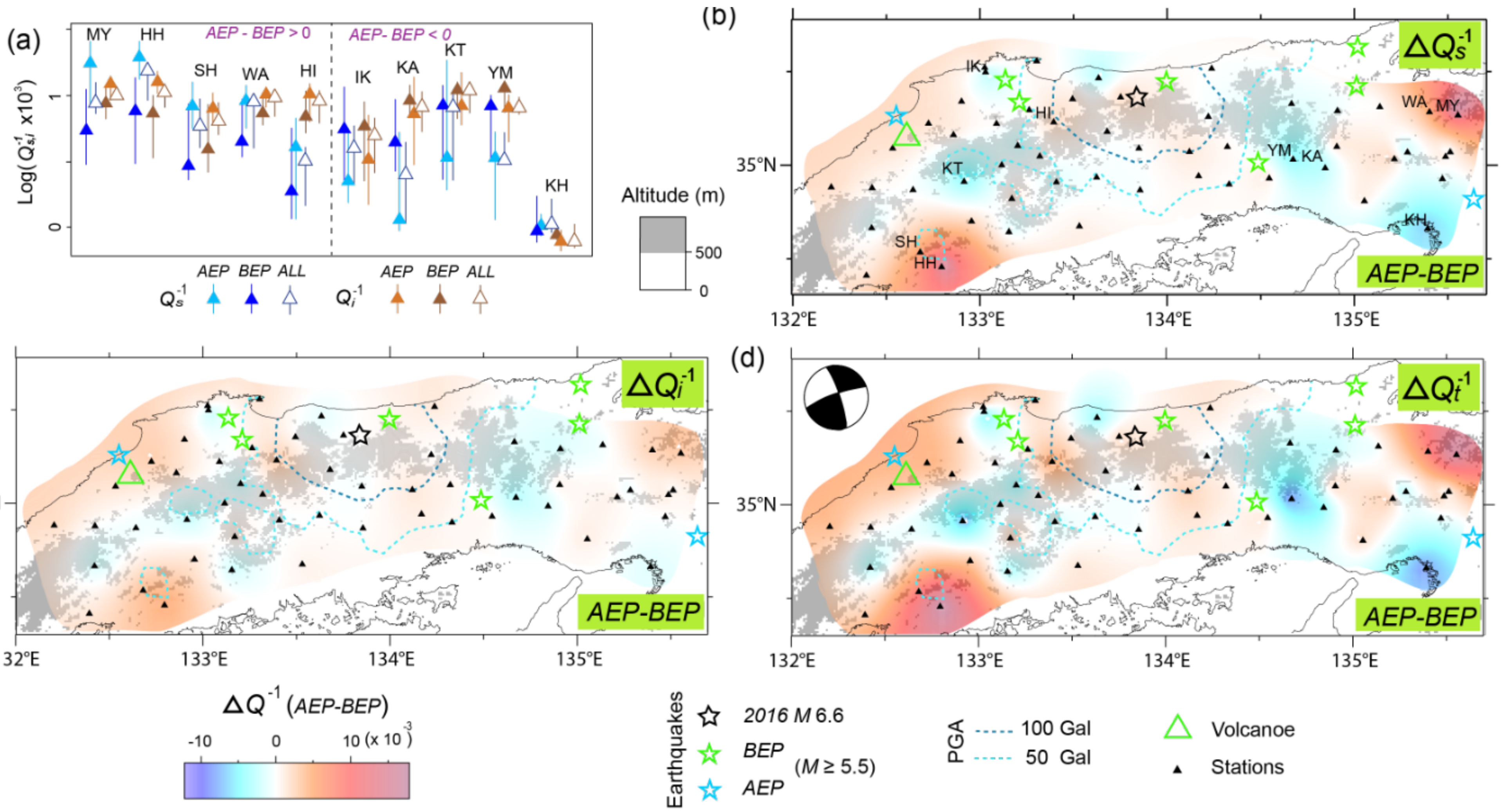

Figure S5. (a) The Q_s^{-1} and Q_i^{-1} values of of the *BEP*, *AEP*, and All (*BEP*+*AEP*) are compared for the stations. (b), (c), and (d) Topographic maps showing the difference between the *BEP* and *AEP* (*AEP-BEP*) for the Q_s^{-1}, Q_i^{-1}, and Q_t^{-1} values, respectively, of the Tottori earthquake at 1.5 Hz. Focal mechanism of mainshock in (d) is from CMT catalog from Hi-net. The styles and symbols are the same as those in Figure 5.
